# Multi-Method Technics and Deep Neural Networks Tools on Board ARGO USV for the Geoarchaeological and Geomorphological Mapping of Coastal Areas: The Case of Puteoli Roman Harbour

**DOI:** 10.3390/s24041090

**Published:** 2024-02-07

**Authors:** Gaia Mattei, Pietro P. C. Aucelli, Angelo Ciaramella, Luigi De Luca, Alberto Greco, Gennaro Mellone, Francesco Peluso, Salvatore Troisi, Gerardo Pappone

**Affiliations:** Department of Science and Technology, Parthenope University of Naples, 80133 Napoli, Italy; gaia.mattei@uniparthenope.it (G.M.); pietro.aucelli@uniparthenope.it (P.P.C.A.); angelo.ciaramella@uniparthenope.it (A.C.); luigi.deluca@uniparthenope.it (L.D.L.); alberto.greco@uniparthenope.it (A.G.); gennaro.mellone1@studenti.uniparthenope.it (G.M.); francesco.peluso@uniparthenope.it (F.P.); gerardo.pappone@uniparthenope.it (G.P.)

**Keywords:** unmanned surface vehicles, artificial intelligence tools, geoacoustic data, photogrammetric models, geomorphological marine surveys, underwater landscape reconstruction

## Abstract

The ARGO-USV (Unmanned Surface Vehicle for ARchaeological GeO-application) is a technological project involving a marine drone aimed at devising an innovative methodology for marine geological and geomorphological investigations in shallow areas, usually considered critical areas to be investigated, with the help of traditional vessels. The methodological approach proposed in this paper has been implemented according to a multimodal mapping technique involving the simultaneous and integrated use of both optical and geoacoustic sensors. This approach has been enriched by tools based on artificial intelligence (AI), specifically intended to be installed onboard the ARGO-USV, aimed at the automatic recognition of submerged targets and the physical characterization of the seabed. This technological project is composed of a main command and control system and a series of dedicated sub-systems successfully tested in different operational scenarios. The ARGO drone is capable of acquiring and storing a considerable amount of georeferenced data during surveys lasting a few hours. The transmission of all acquired data in broadcasting allows the cooperation of a multidisciplinary team of specialists able to analyze specific datasets in real time. These features, together with the use of deep-learning-based modules and special attention to green-compliant construction phases, are the particular aspects that make ARGO-USV a modern and innovative project, aiming to improve the knowledge of wide coastal areas while minimizing the impact on these environments. As a proof-of-concept, we present the extensive mapping and characterization of the seabed from a geoarchaeological survey of the underwater Roman harbor of Puteoli in the Gulf of Naples (Italy), demonstrating that deep learning techniques can work synergistically with seabed mapping methods.

## 1. Introduction

The underwater sectors of coastal areas are naturally dynamic environments, strongly altered by wave action, longshore currents, sedimentary processes and human activity [1,2,3,4,5,6,7]. Along the Mediterranean coasts, these environments often host underwater archaeological sites, representing important evidence of past coastal settlements, nowadays submerged due to the post-glacial relative sea-level rise, often exacerbated by tectonic and volcano-tectonic ground movements [8,9,10,11,12,13]. Documenting these underwater coastal areas is a goal of great scientific interest to understand the responses of natural and anthropic environments to the expected of sea-level rise due to ongoing climatic changes [14,15,16]. However, several decades of underwater research have highlighted the scientific importance of reconstructing the underwater cultural heritage also to plan protection strategies [17,18,19,20]. Recent technological advances have led to increasingly accurate surveys of such complex and dynamic sectors by integrating well-consolidated geo-acoustic methods with remote sensing techniques, in order to collect multidisciplinary and multi-scalar high-resolution datasets [21,22,23,24,25,26,27,28] required to detect and quantify morphological features of both the seafloor and archaeological structures. Geophysical sensors and underwater photogrammetric systems represent a high precision standard for mapping nearshore sectors [29,30,31,32]. The integrated analysis of data acquired by echo-sounders (single-beam and multibeam) and side scan sonars allows not only the processing of high-resolution digital terrain models (DTMs) of the seabed but also the reconstruction of submerged archaeological remains [29,30,31,32]. As far as the photogrammetric systems, 3D or 2D models deriving from photogrammetric data result in high precision reconstructions of ancient anthropic and natural landscapes [33,34,35,36,37]. On the other hand, photogrammetry is a non-invasive technique used for many purposes, such as mapping, industrial applications, cultural heritage research, underwater surveys, visual navigation and geological studies. The success of this measurement technique is due to its reliability and adaptability to multiple scenarios. The principle behind the reconstruction of the three-dimensional model of the object of interest lies in the images of the same object taken from different viewpoints. From knowledge of the geometric parameters of the camera used, it is possible to describe a relationship between the three-dimensional coordinates of the points of the photographed object and the two-dimensional coordinates of the same point on the image [38,39].

In this context, data analysis can be efficiently supported by new tools based on Deep Learning (DL), a branch of Artificial Intelligence (AI) that uses artificial neural networks, structured in multiple “deep” layers, to learn from complex data. It is often used to analyze and recognize patterns in data, such as images, audio or text, and for innovative applications such as object detection [40]. DL is rarely used in marine science despite their potential to process large volumes of data in a short time [41,42,43,44]. On the other hand, marine surveys in coastal areas are a complex issue to be faced by using traditional survey-boats due to shallow waters and the presence of semi-submerged obstacles. This operational complexity increases in the case of geoarchaeological research that typically focus on submerged archaeological structures vulnerable to impacts during the survey and often positioned in legally protected areas [45]. To this end, the open project prototype of a marine drone, namely ARGO-USV (Unmanned Surface Vehicle for ARchaeological GeO-application) was conceived to meet the challenge of efficiently surveying nearshore sectors with submerged and semi-submerged archaeological structures. The scientific challenge is to propose innovative procedures to process high-resolution multidisciplinary datasets in an increasingly efficient way, integrating established methods with Computational Intelligence and Deep Learning based techniques applied to the analysis of data from integrated marine surveys. The three technological pillars of ARGO-USV, aimed at the survey of submerged coastal archaeological heritage are:

1. Application of innovative methodologies in the field of marine coastal surveys;
2. Developing an innovative technology based on multi-method mapping supported by AI modules;
3. Designing and applying a methodology aimed at reducing environmental impact throughout the product life cycle.

This paper presents the technological advances of the ARGO-open-project applied to the integrated survey of a coastal area of cultural value due to the presence of the underwater archaeological remains of the majestic Roman harbour of Puteoli in the Gulf of Naples. The geoarchaeological interpretations are the result of the integration of AI-based tools and established methodologies on board the ARGO-USV.

The rest of the manuscript is outlined as follows. Section 2 shows the motivation behind the construction of ARGO-USV as well as the methodology used to approach the research and the architecture, explained in detail, on which the drone is based. The main modules used and the models trained for deep learning techniques will be presented. Section 3 explains the main techniques used to perform seabed mapping as well as the experiments performed to choose the best classification model. This is followed by Section 4 in which the results obtained both from an archaeological and a technological point of view are discussed. Finally, Section 5 concludes the paper by providing details on the conclusions expressed above and looking at possible future developments.

## 2. Materials and Methods

Wide variety of drones are intensively used for marine research, particularly during oceanographic surveys, environmental surveillance, and monitoring, thanks to the high performance of innovative technologies. Additionally, a new impetus has been given to research in critical areas both in terms of logistics and navigation, due to the reduced dimensions, launching and handling simplicity.

**Figure 1:**
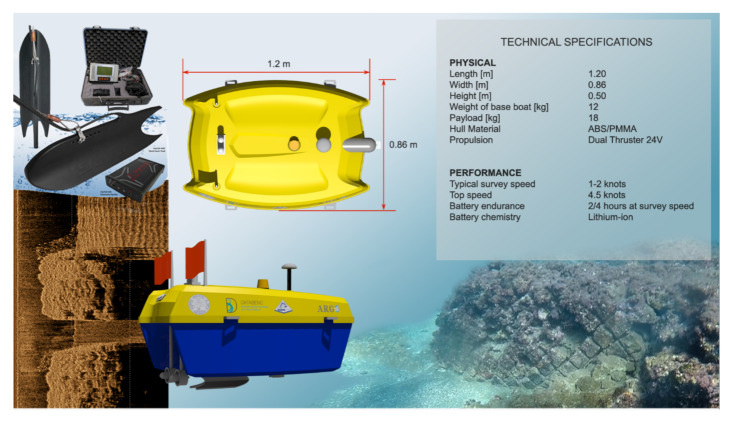
Scheme of ARGO equipment and technical characteristics.

The iMTG (Innovative Marine Technology for Geoarchaeology) laboratory of the Parthenope University has been experimenting with the use of marine drones in the coastal area since 2015, producing several prototypes with which a considerable amount of scientific data and operational experience has been gathered [46]. In light of this background, ARGO-USV (Argo-Hellenic-USV, Attiki, Greece) has been designed and engineered in 2020 for carrying out integrated surveys of coastal areas. It is a marine drone capable of operating efficiently in both remote and automated modes and equipped with geoacoustic and optical sensors interfaced with AI tools (Figure 1).

The system is based on two integrated sub-systems: an unmanned surface vehicle (USV) and a ground control station (GCS).

### 2.1. USV

The unmanned surface vehicle ARGO has been optimized to carry out morpho-bathymetric surveys in very shallow water sectors (0–25 m). The project was created to satisfy the requirements necessary for the mission: good maneuverability at low speeds; a load capacity capable of accommodating the necessary equipment; ease of transport and placement into the water (launching); autonomy of at least 2–4 h; and the sharing of data acquired in real time with the GCS. These needs led to the creation of an original project not derived from commercial hulls, with a catamaran structure 0.86 m wide and 1.20 m long. The hull is made of ABS + PMMA (Acrylonitrile Butadiene Styrene + PolyMethyl MethAcrylate), which is a polymeric plastic based on styrene. This material not only guarantees characteristics of resistance, mechanical rigidity, and corrosion resistance but can also be used through an industrial process called vacuum thermoforming, a technique widely used in the automotive industrial sector, capable of producing small series of very resistant and light objects. The architectural conformation of the catamaran hull was conceived to create a large and regular central space for the payload and further space in the lateral hulls for battery positioning. For the motorization, two robust brushless electric outrunner thrusters (equipped with left-handed and right-handed propellers: model T-200 produced by the US company BlueRobotics (2740 California St, Torrance, CA 90503, USA)) were used. This model can guarantee a maximum thrust (full throttle FWD/REV thrust nominal 16 V) of 5.25/4.1 kg such as to allow the ARGO-USV a survey speed of 1/2 knots and a maximum transfer speed of 4 knots.

**Figure 2:**
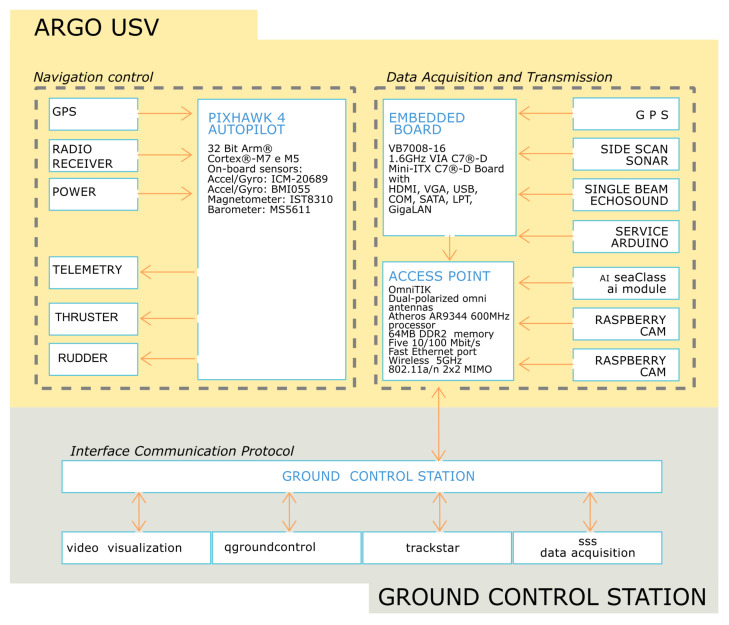
ARGO-USV architecture.

The overall architecture of this work (Figure 2) can be divided into two main sections. The first (yellow) is the architecture developed onboard the USV. It encapsulates the *command and control system*, while the other section is the one that interacts remotely with the *ground control station* (gray). The *command and control system* has two different blocks. The first is dedicated to the guidance and navigation control of the drone, namely the *flight controller*, while the second is dedicated to sensor management and the sharing of acquired data with the ground control station via telemetry, named *management and data acquisition*.

The ARGO-USV is able to operate both in autonomous mode, according to a previously planned navigation plan, and in unmanned mode, manually controlled by radio control. Navigation is managed by a Pixhawk 4^®^ (Holybro, San Diego, CA 92111, USA) an advanced autopilot designed and created in collaboration with Holybro^®^ (San Diego, CA 92111, USA) and the PX4 team. This hardware uses the PX4 operating system, used in both commercial and academic fields thanks to its open features (open standard). Additionally, a global community of users and developers allows software customization. For the same reason, the open-source and multiplatform software QGroundControl (http://qgroundcontrol.com/ accessed on 29 January 2024) was adopted to manage and plan the survey. In this case, the customizable platform is also supported by a large community of developers. The result is the satisfying integration of the external hardware and software modules.

The **navigation controller** block is based on a flight controller, the Pixhawk 4, coupled with the QGroundControl mission planning and management software. Both applications are off-the-shelf components with open-source features, which allow customization and integration with external software and hardware modules by modifying the source code. In particular, the first block includes the flight controller Pixhawk4 and the PM04 electrical power system and associated subsystems consisting of rudder actuators, servo-controls, thrusters and an 8-channel radio control (RC) receiver. The Pixhawk 4^®^ is an autopilot based on the Pixhawk project FMUv5 open hardware design and runs PX4 on the NuttX OS.

The **management and data acquisition** block is a proprietary iMTG Lab system and is based on an embedded PC, the VIA VB7008 (VIA Technologies, Inc. 940 Mission Ct, Fremont, CA 94539, USA), dedicated to the management and data acquisition of the main sensors (SBES, SSS). The VIA VB7008 is based on the processor 1.6 GHz VIA C7^®^-D and features 8 GB of RAM, a 500 GB hard disk and numerous I/O comm ports. The proprietary system installed on the VIA VB7008 Embedded PC guarantees the coexistence between the different sensors and subsystems required for the correct execution of the mission, integrating them into a single database and transmitting the data to the Ground Control Station (GCS) via LAN. For security reasons, the data generated by each sensor is also stored in bulk on a mass storage device onboard the USV.

This block is connected—via the numerous I/O ports—with the GPS positioning system, IMU attitude sensors, SBES, SSS, video cameras (above and below sea level) and a series of subsystems developed by the iMTG-lab (based on Raspberry microprocessors and Arduino microcontrollers) that support onboard activities. In detail, the subsystems include an ultrasound-based obstacle detection system, a temperature system, a photogrammetric camera synchronization device, and a trim-control system, already installed on a previous version of this technological project.

Special attention was paid to the acquisition of bathymetric data, to which two subsystems were dedicated. The first one verifies that the angle between the echo sounder axis and the horizon does not exceed a predefined threshold during the planning phase, using attitude sensors. The second automatically calculates the draught of the SBES transducer in relation to the water surface using ultrasonic sensors. The draught can vary due to different combinations of weight and/or payload, due to the modular configuration of the ARGO-UVS.

This feature was designed and implemented because the project involves a multi-mission configuration with different combinations of sensors and their batteries. These different combinations can result in different weights of the USV and consequently different distances from the sounder transducer to the water surface. Finally, all acquired data are appropriately formatted, shared and made available via a 5 GHz broadband local area network based on long-range WiFi transmitters with an operative range of 1 km. For this purpose, the MikroTik OmniTIK (I-NetPartner GmbH Online Services, Fraunhoferstraße 4, D-73037 Goeppingen, Germany) is used, a weatherproof outdoor access point with dual polarization Omni antennas for other standard 5 GHz 802.11a/n devices. It features five Ethernet ports, PoE support and a powerful integrated 802.11a/n wireless radio with an aggregate throughput of up to 200 Mbits. This bandwidth allows all data to be sent to the GCS in broadcasting mode, thus allowing different devices to connect simultaneously, guaranteeing the multidisciplinary use of the acquired data. The ARGO-USV is an experimental system and can be equipped with different sensor configurations, including acoustical and/or optical instruments. In detail, in this study, the configuration used was the following:

- A single-beam digital echo sounder for bathymetric measurements with a frequency of 200 KHz;
- A side scan sonar for areal geomorphological investigations with an operating frequency of 450 KHz;
- A photogrammetric system with 3 high-definition cameras positioned on different axes;
- A GPS positioning system.

The single-beam digital echo sounder (SBES) is a 200 kHz Ohmex instrument with a maximum acquisition depth of 60 m, therefore optimized for coastal bathymetric measurements. The Tritech Side-Scan StarFish 450C Sonar (Mariscope, Redderkoppel 6A, 24159 Kiel, Germany) is a small instrument (0.378 m long), optimized for coastal waters (CHIRP transmission at 450 KHz), with a maximum resolution of 0.0245 m (1 inch) and a slant range between 15 and 100 m. The small dimensions of the vessel allowed the GPS and acoustic transducers to be appropriately positioned, reducing to a few cm any offsets, which are properly corrected during the post-processing. To integrate the information obtained from the geophysical sensors (SBEAM and side scan sonar), the USV is also equipped with a photogrammetric system based on three underwater cameras. The photogrammetric system is based on two side-by-side Xiaomi YI Action cameras nadirally positioned and a GoPro Hero 3 (GoPro Inc, Beijing 100000, China) tilted at an angle of 30 degrees. The nadiral cameras form a stereoscopic base (b), which can be varied in relation to the bathymetric range of the study area, guaranteeing a minimum overlap of 80%. The third camera (GoPro Hero 3), with a different shooting angle, guarantees coverage of non-covered sectors. The post-processing algorithm used to reconstruct the three-dimensional models of the archaeological structures of interest is the Structure from Motion (SfM) algorithm, implemented in the main close-range photogrammetry software. It is a photogrammetric imaging technique for the estimation of three-dimensional structures from sequences of images obtained from different viewpoints that can be obtained from a moving camera. The platform equipment, the installation of the command and control system, the construction of the subsystems and the payload interface were entirely designed and engineered at the iMTG Lab of Parthenope University.

### 2.2. Ground Control Station (GCS)

All data acquired by the USV are transmitted to the ground control station via a 5 GHz broadband LAN. A series of workstations are connected to this network, generally based on laptops and/or portable devices such as tablets and smartphones. From the perspective of multidisciplinary research, there are several workstations, namely

- A workstation dedicated to navigation (QGroundControl);
- A workstation for data acquisition (TrackStar);
- A workstation for side scan sonar data acquisition;
- Several portable devices, dedicated to the visualization of the emerged and submerged onboard video cameras.

### 2.3. Multi-Method Approaches

ARGO-USV applies a multimodal mapping technique to data acquisition, which involves the simultaneous use of different kinds of onboard sensors, intending to locate, map and store information on submerged geoarchaeological structures and seabed morphology 198 related to the study area Figure.

**Figure 3:**
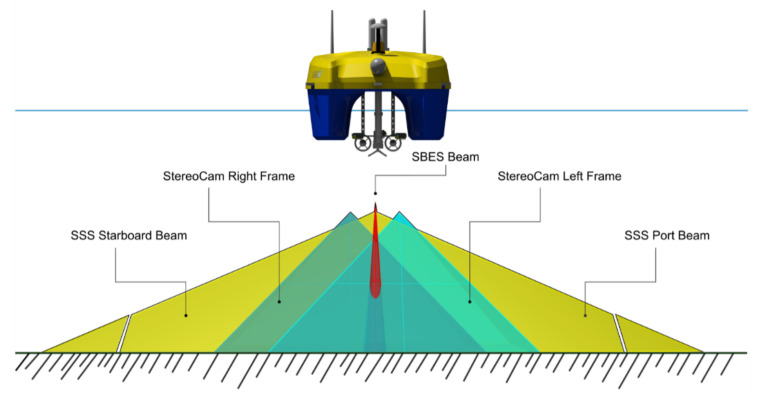
The multimodal approach to a multi-scale integrated survey of shallow water sectors.

The multimodal mapping of the study area (Puteoli Roman port) was obtained by integrating the processing of a multidisciplinary and multi-scale dataset (Figure 3). The acquired data can be divided into large- and small-scale measurements.

Small-scale measurements performed by geophysical sensors (Single Beam Echo Sounder and Side Scan Sonar) allow the extensive mapping of the seabed, its geomorphological characterization, and the identification of possible archaeological structures lying on it. Large-scale measurements obtained by the underwater photogrammetric system allow the creation of three-dimensional models of the underwater landscape, as well as the submersion of significant targets (such as creperies, harbor piers, wall structures, etc).

**Figure 4:**
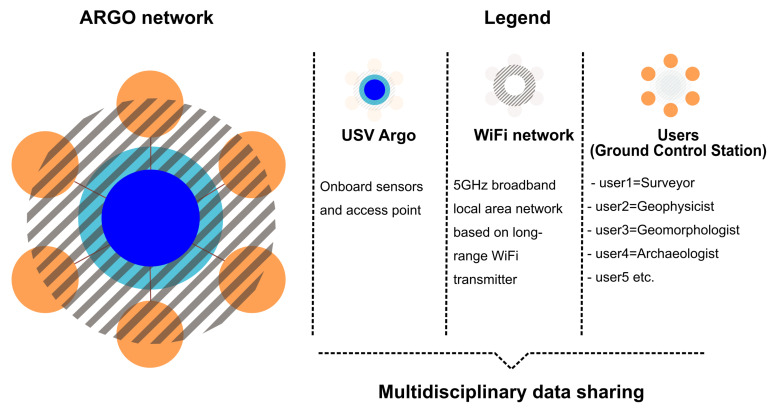
Conceptual model of data sharing during the multidisciplinary surveys carried out by ARGO drone.

Additionally, a DL-based module has been programmed to automatically characterize the seabed (discriminating between the soft bottom and rocky/archaeological one) by analyzing, in post-processing, the videos of the onboard underwater cameras (Computational Intelligence & Smart Systems Lab of Parthenope University). The use of this technique, along with the data sharing between the various surveyors, guarantees the effective reconstruction of submerged environments and geoarchaeological targets (Figure 4).

### 2.4. AI Tools

**Figure 5:**
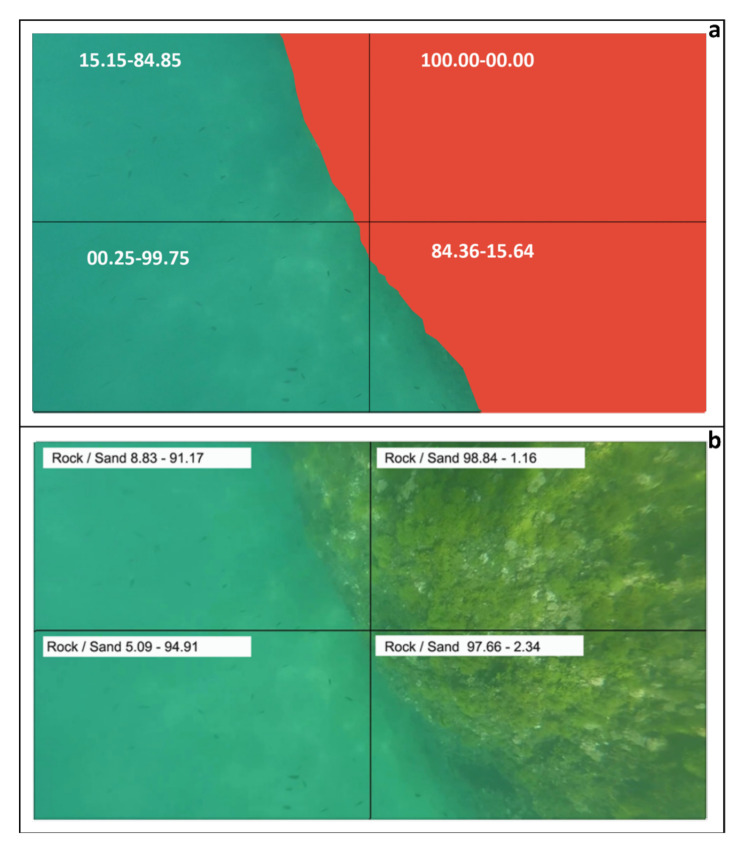
(**a**) Frame 1 extracted from the CLIP-A film and assessed by expert judgement to classify the areas according to *rock* class (red background) and *sand* class; (**b**) percentage of *rock* and *sand* classes detected by aiSeaClass module using Model 4.

The AI module called aiSeaClass (Artificial Intelligence Seabed Classification) is an application capable of characterizing the seabed using DL techniques by analyzing data from the onboard underwater cameras. The information produced by this module is complementary to those acquired by the other onboard sensors (side-scan sonar, digital echosounder, photogrammetric system). However, the challenge of this experimental application is to propose a totally passive technique of surveying. This experimental tool characterizes the underwater environment into two classes, namely *Sand Bottom* and *Rock Bottom*, representing respectively the type of seabed. This binary classification does not accurately classify the seabed, as it does not take into account intermediate configurations or the presence of other morphologies such as vegetation. The aiSeaClass experimental system consists of a Convolutional Neural Network (CNN), which enables the classification of the images acquired by the USV into two classes: rocky/anthropic and soft bottom. A total of 4 models, using different configurations, have been trained on a dataset of 498 images previously acquired by the marine drones of the iMTG Laboratory during marine research in recent years. The system has been developed by testing different CNN configurations, which vary in both structural and parametric terms, but with the same type of layers typical of neural networks (see Table 1). The user interface of this application splits the video screen into four quadrants (Figure 5 calculating the percentage of each class in each quadrant. The percentages are calculated for each frame and therefore, taking into account the rate of 30 fps (frames per second) the data is updated on the screen every 1/30 of a second. Finally, the module computes an (x,y,z) file where (x,y) is the horizontal position of the center of each frame and *z* is the overall percentage of rock.

### 2.5. Tests of AI Tool

This experimental module was conceived in the context of geomorphological coastal surveys without acoustic wave emissions. In detail, we developed and trained 4 different CNNs able to identify autonomously the percentages of rocky and soft bottom present in each frame of the acquired video (see Section 2).

The model was obtained by testing different configurations and parameters.

The analyzed video (CLIP-A) included a total of 2430 frames, expressing a quantitative value for each frame acquired. CLIP-A, filmed in the study area, shows the ’flyover’ of two ancient Roman port structures (pilae) made of hydraulic concrete and measuring approximately 5 m × 5 m. The technical data provided by the analyses described in Section 2.3 allow an initial evaluation of the efficiency of this application. The graphically modified frames (Figure 5a) were analyzed to evaluate the total number of pixels per category. This ratio represented the comparison value against those provided by the AI-based system for each model, regarding the same frame. The frame submitted to the Expert Judgement (relative to time 00:13 of the Clip-A) was chosen due to the presence of both types of morphologies.

In Figure 5a, the labels in white refer to the percentages assigned respectively to the Rock class and the Sand class by the Expert Judgement. Similarly, in Figure 5b white labels refer to the estimated percentages from the AI module.

### 2.6. Post-Processing and Data Elaboration

The multimodal mapping of the study area (Puteoli Roman port) was obtained by integrating the processing of a multidisciplinary and multi-scale dataset. The bathymetric system was used to measure the depth of the archaeological remains and to reconstruct the detailed bathymetry of the seabed. The depths (D) were referred to as the mean sea level (MSL), correcting each measurement (M) with respect to the **tidal** level (IL) obtained from the Naples tide gauge of the National Mareographic System due to its proximity to the area of investigation:

(1) D=M+IL

Finally, the depth measurement was corrected concerning the transducer submersion, measured by the acoustic system for the draught measuring. The bathymetric data were processed in a GIS environment to reconstruct the seabed morphology, as described in Section 3. The morpho-acoustic mapping of the coastal sector was obtained by a side scan sonar system optimized to be installed on small USVs and capable of identifying seabed morphology (sandy, rocky, muddy, etc.), by discriminating any archaeological targets. During the survey, several acoustic acquisitions were carried out with different lateral beam openings. Having evaluated the results and the morphological characteristics of the seabed as well as the bathymetric depth, a slant range of 30 meters with an overlap of 50% was selected as the optimal setting. The sonographs were post-processed using Chesapeake Sonar Web Pro 3.16 software to create a single GeoTIFF mosaic, by obtaining a sonar coverage of the study area. A further post-processing phase concerned the analysis of the backscattering signal to discriminate between geoarchaeological targets, sandy sectors and rocky ones. The Time-Varying Gain (TVG) filter was also used at this stage, which emphasizes the gain for the acoustic signals reflected from the structures positioned at the edges of the sonographs. The photogrammetric survey was carried out simultaneously with the acoustic one, by using the photogrammetric system installed on board. Before the survey, the calibration of the two Xiaomi cameras in an underwater environment close to the study area was performed to achieve the inner orientation parameters. During the survey, the two cameras were synchronized using the onboard trigger system. The photogrammetric 3D model was calculated as follows. Firstly, the alignment procedure of the images was performed by Agisoft Photoscan software (https://www.agisoft.com/ accessed on 29 January 2024). Secondly, a dense point cloud was extracted and georeferenced by using the coordinates of the ground control points (GCPs) determined by GPS Fast static procedure. Finally, the 11.556.269. cloud was exported in GIS format using the open-source software CloudCompare (https://www.danielgm.net/cc/ accessed on 29 January 2024). The so - so-obtained point cloud was interpolated by a Topo-to-Raster interpolator, producing a DTM with a grid of 0.09 × 0.09 m.

## 3. Results

In this section, a comprehensive explanation of the main techniques used to conduct seafloor mapping will be provided. This includes a detailed exposition of the methodologies adopted to acquire data on seafloor topography and seafloor composition. The experiments conducted to select the most suitable classification model and to validate it with other seabed mapping methods are also discussed in detail.

### 3.1. Study Area—Puteoli Roman Port

**Figure 6:**
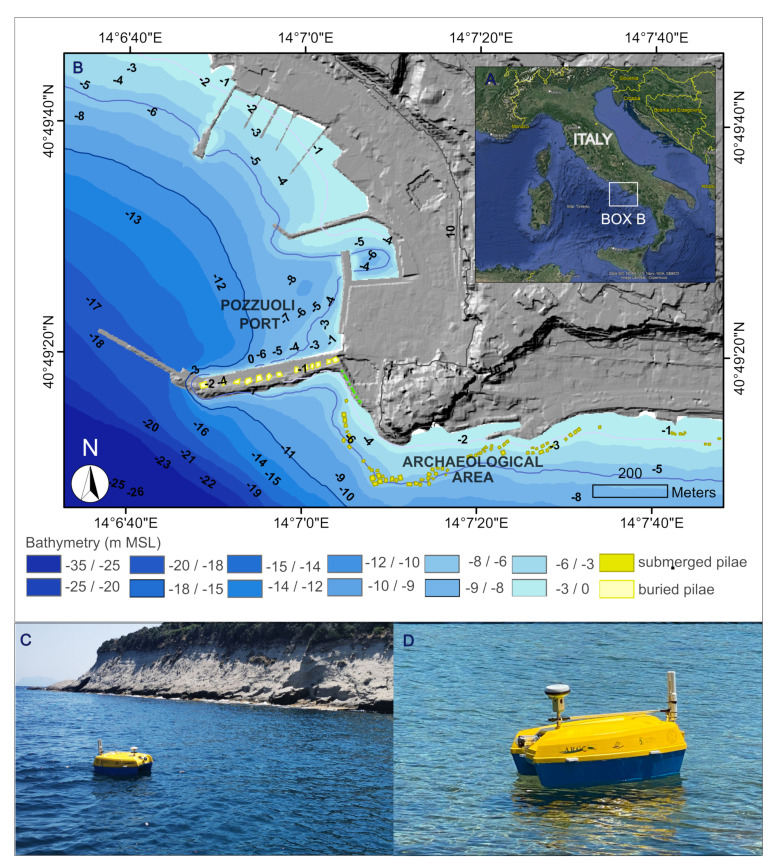
(**A**) Location map; (**B**) study area; (**C**,**D**) ARGO-USV in action.

An integrated marine survey of the underwater Roman harbor of Puteoli along the Pozzuoli coast was carried out by the ARGO-USV (Figure 6), leading to the extensive mapping and characterization of the seabed and the 3D model reconstruction of some archaeological structures. This archaeological site was chosen as it is the best-preserved and most magnificent port work in the Gulf of Naples (Southern Italy), built during the Roman period (Sommella, 1980; Camodeca 1994).

In fact, in those years, the Roma’s granary supply represented an important commercial requirement. However, while the Ostia port was much exposed to storms, making the long stop of large merchant ships dangerous, Puteoli Port was much safer for the storage of large quantities of Egyptian or African grain. The harbour system was supported by arches mounted on concrete pillars made of the hydraulic form technique (pilae), experimented and largely used in the Phlegrean area as described by Vitruvius. The remains of the main pier, known as Molo Caligoliano because of the traditional attribution of the ruins to the impressive bridge made by Emperor Caligula (37–41 AD) from Puteoli to Baiae, according to Svetonius, are nowadays completely buried by modern renovations of Pozzuoli Port (Figure 6).

**Figure 7:**
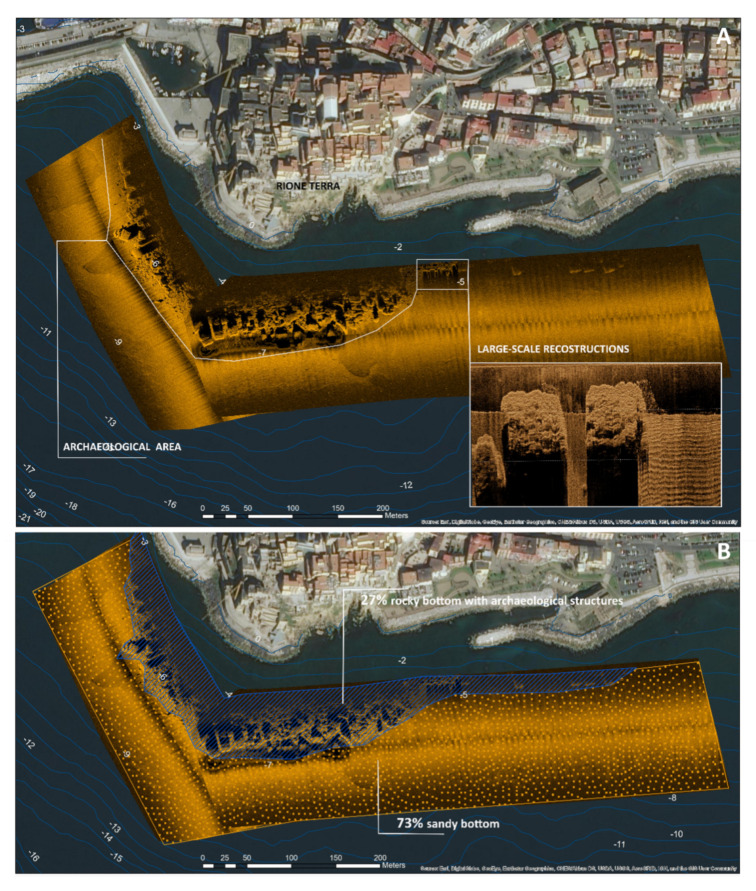
(**A**) SSS mapping of the underwater archaeological area; (**B**) sea bottom characterization from SSS signal analysis.

However, the underwater area, the focus of the presented survey, is characterized by numerous submerged squared structures (pilae) composed of opus caementicium (Roman concrete). This imposing series of robust and irregularly spaced pilae has been interpreted as a coast protection system that was built at a certain distance from the coast in order to reduce the wave action; according to Camodeca (1994), they were probably constructed under Emperor Augustus (31 BC–14 AD). Altogether, they form an almost continuous line, which extends for about 1 km sub-parallel to the modern coastline, from which they are 40 to 150 m distant (Figure 7).

### 3.2. Multi-Scale Seabed Mapping

The overlay between the small-scale mapping of morpho-acoustic elaborations and the large-scale mapping of video-photographic data allowed the efficient GIS reconstruction of the underwater morphologies.

**Figure 8:**
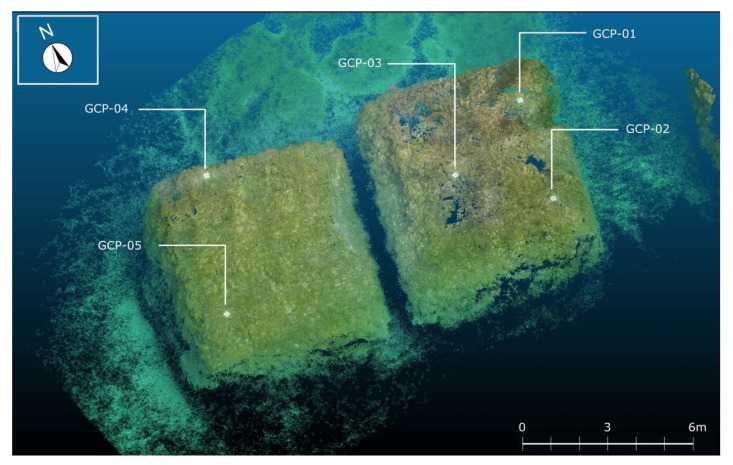
Photogrammetric point cloud with ground control points (GCP).

In particular, the small-scale mapping of the underwater area was obtained from the elaboration of SSS data, whose interpretations favored the classification of different types of seabed morphologies, discriminating between submerged landforms and archaeological structures like pilae (Figure 7). If 73% of the investigated area is characterized by a sandy bottom, 27% is a rocky bottom with archaeological structures. It is worth noting that almost all the pilae lie near the edge of a submerged terrace at a depth of 5 m BSL. Each pila has a squared base of 7 by 6 m and a height of 4.0 to 4.5 m, although there are some pilae with a base of 10 m. However, it was difficult to discriminate the bottom typology in the areas nearest the archaeological structures, due to the acoustic shadows created by the 4-meter elevated anthropic structures. For this reason, a large-scale analysis was applied to three pilae, probably aligned to compose a pier, in the eastern sector of the archaeological area, in order to both classify the sea bottom nearest the archaeological structures and obtain the 3D photogrammetric model of such structures (Figure 8). The coordinates of the Ground Control Points used to georeference the 3D photogrammetric model were determined by processing dual-frequency GNSS data (GPS + Glonass) collected in fast static mode from a receiver positioned on a 2.5-metre-high pole held vertically by an underwater operator. Using a reference station positioned at a distance of approximately 10 km, fixed solutions were determined for three GCPs and floating solutions for the other two. The standard deviations of the coordinates obtained with fixed solutions are approximately 2 cm, and those for floating solutions amount to 15 cm. The fixed solutions were inserted within the photogrammetric block as Ground Control Points, while the float solutions were used as CheckPoints. Downstream of the photogrammetric adjustment process, the residuals on the GCPs were approximately 6 cm in planimetry and 1 cm in altimetry, while for the CheckPoints these residuals were just under 20 cm in planimetry and 6 cm in altimetry.

**Figure 9:**
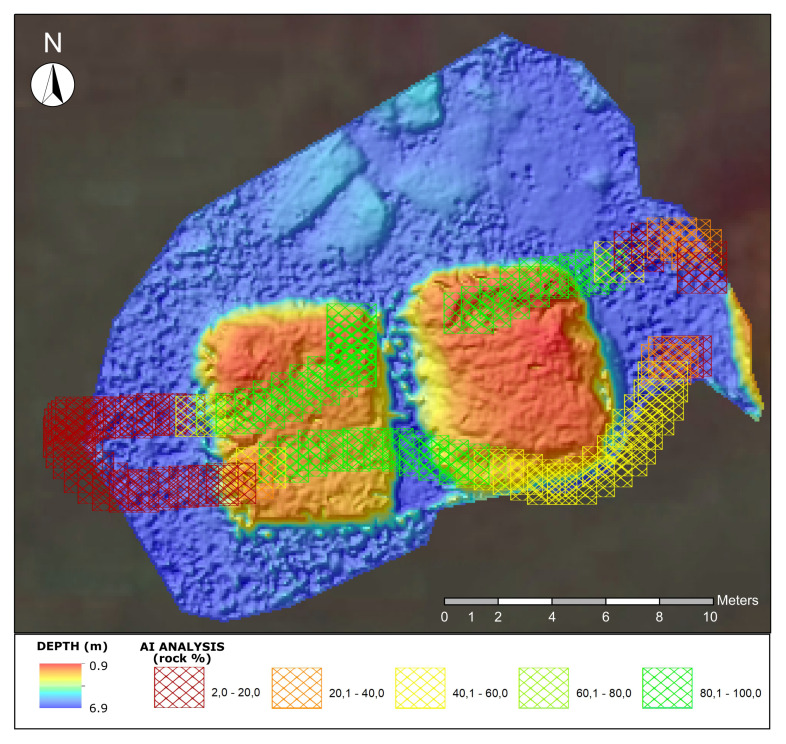
Complete ARGO-USV route processed with the aiSeaClass module using Model 4 and colored according to the presence (%) of rock, overlaid with the photogrammetric 3D model.

The high-resolution 3D model (Figure 9) obtained by interpolating the photogrammetric point cloud allowed the detailed reconstruction of these structures, with a cell resolution of 0.09 × 0.09 m and the following altitude statistics:

- min value −6.88 m;
- max value −0.96 m;
- mean value −4.57 m;
- standard deviation 1.85 m.

### 3.3. Seabed Mapping Using Deep Learning

In the same area where the 3D photogrammetric model was reconstructed (Figure 8), the aiSeaClass module was tested to characterize seabed morphologies and submerged archaeological structures (i.e., the detected pilae) “flown over” by the drone using the onboard underwater video cameras.

In fact, the rock class includes archaeological structures built in concrete like pilae. Accordingly, the class 80–100% rock fits with the area where the pilae are located.

Nevertheless, the application represents the first stage of a research project aimed at assessing the feasibility of applying AI techniques in this difficult operational theatre. Despite this, interesting results emerged from this application, which allows for obtaining information on the area under investigation in a totally passive mode and synergy with the other sensors installed on the drone.

Table 2 shows the percentage of the rock class estimated by the different CNN models for each quadrant of Frame 1. Model 4 was selected as the most congruent with the expert judgment and, consequently, the (x,y,z) file with the overall percentage of rock per frame was exported. The results from the application of Model 4 were subsequently overlaid with data obtained through photogrammetric and geoacoustic techniques. This overlay was performed to validate not only the specific predictions of Model 4 but also, in a broader sense, the aiSeaClass module as a whole. The integration of the photogrammetric and geoacoustic results, in Figure 9, allows for cross-validation, making it possible to assess the consistency and reliability of the predictions provided by Model 4 and to extend this validation to the broader context of the application of the aiSeaClass module as a whole. This multifactorial approach reinforces the robustness of the conclusions drawn and helps to consolidate the overall accuracy of the aiSeaClass underwater classification system.

## 4. Discussion

The experimental results, shown in Figure 9, demonstrate the efficiency of a multiscale and multi-technic approach to the marine geoarchaeological surveys of coastal areas with indirect methods. The small-scale survey allowed an extensive mapping of the underwater sector and the detection of the archaeological area as a first approximation. The large-scale data elaboration applied to those sectors where the archaeological remains have been detected allowed coupling a detailed 3D reconstruction of the archaeological structures with a seabed characterization through the experimental AI module.

From an archaeological point of view, we interpreted this imposing series of robust and irregularly spaced pilae as a coast protection system that was built at a certain distance from the coast to reduce the wave action. According to Camodeca (1994), that long breakwater system was constructed in the same period (under Emperor Augustus, 31 BC–14 AD) when other similar coastal structures were created along the coast of Campi Flegrei and the port of Pozzuoli itself was restored and equipped with a 372 m long pier (the so-called Molo Caligoliano). More in general, the game-changer in the field of geoarchaeological surveys certainly is the high-resolution, extensive reconstruction of underwater sectors and archaeological structures intended as the witness of the ancient anthropized coastal landscape.

According to the first pillar of the ARGO project, the proposed technological procedures facilitated the investigation of critical coastal sectors such as that of Puteoli submerged harbor, which was so far prohibited to traditional survey boats due to shallow depth and presence of outcropping natural and anthropic semi-submerged structures. The methodology allowed for the acquisition of large amounts of georeferenced data, which favored the geoarchaeological and geomorphological interpretation in the study area.

Following the second pillar of the ARGO project, the integration of different typologies of sensors allowed a multimodal acquisition of different kinds of data, the fusion of which produced a high-resolution reconstruction of the investigated area. The onsite experiences with ARGO-USV produced a multi-scale mapping of the surveyed area coupling the extensive characterization of the seabed with the 3D reconstruction of some archaeological structures of high scientific and cultural value.

Concerning the AI tool, in particular, the module implemented for analyzing the information coming from the onboard video cameras not only allowed the characterization of the seabed nearby of the 3D-reconstructed archaeological structures but also supplied such information in a completely passive mode, contributing to a more sustainable marine survey. To this end, additional object detection and classification tools, built in the iMTG laboratory, in partnership with the Computational Intelligence & Smart Systems (CI&SS) and NEPTUN-IA (Multidisciplinary Research Laboratory for the Artificial Intelligence at the Sea—UniParthenope), have been implemented.

Regarding the third pillar of the ARGO project, strong attention was paid to environmental issues and related impacts. The drone was designed using recyclable or recycled construction materials, in line with the *Three Rs rule* (the three Rs are defined as Reduce, Reuse and Recycle). Many structural elements, such as the hull or the aluminum of the sensor support tubes and the steel of the thruster brackets, are made from recyclable parts that can be recycled at the end of their operational life. In addition, several recycled elements such as the sensor support plane (made of wood) and the electronics support made of forex, ex-advertising panels were used. Even in operations management, an attempt was made to limit the possible environmental impact by adopting low-power electric motors. Finally, regarding the critical aspects, the multimodal payload chosen for ARGO technological project required careful navigation planning due to the different footprints of the onboard sensors [47,48,49]. The post-processing of the whole dataset coming from geoacoustic, optical and AI sensors integrated with data from detailed direct surveys, led to the precise mapping of the coastal landscape in terms of both seabed classification and reconstruction of the main underwater archaeological structures detected during the survey.

## 5. Conclusions

Technological progress related to hardware miniaturization and personalized software opened new scenarios for coastal geoarchaeological studies. One of the main targets for future development is to integrate AI systems for processing data acquired in real-time mode. From this perspective, we have developed a platform that can process data in real time and facilitate survey management [50]. This platform will be installed on ARGO-USV to improve its data processing performance and offer a real-time service. The integrated use of aerial and marine drones equipped with sophisticated instrumentation such as LIDAR, multi-beam, side-scan-sonar, and photogrammetric sensors coupled with the use of new methodologies supported by Artificial Intelligence and Machine Learning modules is allowing the realization of surveys in critical shallow water sectors, unthinkable until a few years ago. However, the growing integration between different instrumentation, experiences and knowledge, along with the always-indispensable direct surveys, is enabling the scientific community not only to deal with the complex issues related to the survey of underwater landscapes but also to increase the awareness in perceiving them, opening a very interesting window of opportunity for marine and coastal research. In the future, we want to focus even more on the development of totally passive, acoustic-emission-free and therefore near-zero-impact surveying methods for the marine environment, implementing object detection and classification tools capable of classifying different kinds of objects (e.g., rocks, sand, anthropic structures, litter, etc.) based on pre-processing and data integration methodologies, in order to obtain more complex and detailed digital documentation of the underwater coastal landscapes with archaeological value. Additionally, we are testing a new AI-based system capable of detecting and georeferencing in real-time the GCPs (Ground Control Points) in order to quickly build high-resolution photogrammetric models of underwater key sites. This technological project aimed to integrate well-consolidated methods with innovative techniques in order to obtain an efficient approach to the high-resolution reconstruction of natural and anthropic submerged landscapes.

## Figures and Tables

**Table 1:** Models Configuration - Configuration of parameters and number of layers of the models.

Parameters	Model 1	Model 2	Model 3	Model 4
Learning rate	3 × 10^−6^	3 × 10^−6^	1 × 10^−6^	1 × 10^−5^
**N. Layers**	**Model 1**	**Model 2**	**Model 3**	**Model 4**
Convolutional with dropout (*p* = 0.2)	5	6	5	6
MaxPooling	5	6	6	5
Flatten	1	1	1	1
Dense	2	2	2	2

**Table 2:** Frame 1—rock percentage estimation using four CNN models and comparison with expert judgment.

	Quadrant 1 (%)	Quadrant 2 (%)	Quadrant 3 (%)	Quadrant 4 (%)	RMS Deviation
**Expert Judgement**	**15.15**	**0.00**	**84.36**	**100.00**	**-**
**Model 1**	6.36	4.53	95.27	97.23	7.40
**Model 2**	13.43	7.83	96.92	98.39	7.49
**Model 3**	8.83	5.09	97.66	98.84	7.83
**Model 4**	11.97	9.07	91.46	93.33	6.90

## Data Availability

The datasets generated during and/or analyzed during the current study are not available in a repository as they are described in the text but are available from the corresponding author on reasonable request.

## Abbreviations

The following abbreviations are used in this manuscript:

USV	Unmannned Surface Vehicle
ARGO-USV	Unmannned Surface Vehicle for ARchaeological GeO-application
GCS	Ground Control Station
AI	Artificial Intelligence
DL	Deep Learning
iMTG	Innovative Marine Technology for Geoarchaeology
LIDAR	Laser Imaging Detection and Ranging
GPS	Global Positioning System
SBES	Single-Beam Echo Sounder
SSS	Side Scan Sonar
GCP	Ground Control Point

## References

[1] Trenhaile A.S. (1997). Coastal Dynamics and Landforms.

[2] Woodroffe C.D. (2002). Coasts: Form, Process and Evolution.

[3] Anfuso G., Dominguez L., Gracia F. (2007). Short and medium-term evolution of a coastal sector in Cadiz, SW Spain. Catena.

[4] Ferrando I., Brandolini P., Federici B., Lucarelli A., Sguerso D., Morelli D., Corradi N. (2021). Coastal modification in relation to sea storm effects: Application of 3D remote sensing survey in Sanremo Marina (Liguria, NW Italy). Water.

[5] Martínez del Pozo J., Anfuso G. (2008). Spatial approach to medium-term coastal evolution in south Sicily (Italy): Implications for coastal erosion management. J. Coast. Res..

[6] Molina R., Manno G., Lo Re C., Anfuso G., Ciraolo G. (2020). A methodological approach to determine sound response modalities to coastal erosion processes in Mediterranean Andalusia (Spain). J. Mar. Sci. Eng..

[7] Pourkerman M., Marriner N., Morhange C., Djamali M., Amjadi S., Lahijani H., Beni A.N., Vacchi M., Tofighian H., Shah-Hoesseini M. (2018). Tracking shoreline erosion of “at risk” coastal archaeology: The example of ancient Siraf (Iran, Persian Gulf). Appl. Geogr..

[8] Pourkerman M., Marriner N., Morhange C., Djamali M., Lahijani H., Amjadi S., Vacchi M., Jelodar M.E., Spada G., Tofighian H. (2021). Late Holocene relative sea-level fluctuations and crustal mobility at Bataneh (Najirum) archaeological site, Persian Gulf, Iran. Geoarchaeology.

[9] Vacchi M., Ghilardi M., Stocchi P., Furlani S., Rossi V., Buosi C., Rovere A., De Muro S. (2020). Driving mechanisms of Holocene coastal evolution in the Bonifacio Strait (Western Mediterranean). Mar. Geol..

[10] Vacchi M., Joyse K.M., Kopp R.E., Marriner N., Kaniewski D., Rovere A. (2021). Climate pacing of millennial sea-level change variability in the central and western Mediterranean. Nat. Commun..

[11] Caporizzo C., Gracia F., Aucelli P., Barbero L., Martín-Puertas C., Lagóstena L., Ruiz J., Alonso C., Mattei G., Galán-Ruffoni I. (2021). Late-Holocene evolution of the Northern Bay of Cádiz from geomorphological, stratigraphic and archaeological data. Quat. Int..

[12] Aucelli P.P., Mattei G., Caporizzo C., Cinque A., Amato L., Stefanile M., Pappone G. (2021). Multi-proxy analysis of relative sea-level and paleoshoreline changes during the last 2300 years in the Campi Flegrei caldera, Southern Italy. Quat. Int..

[13] Amato V., Aucelli P.P., Mattei G., Pennetta M., Rizzo A., Rosskopf C.M., Schiattarella M. (2018). A geodatabase of Late Pleistocene-Holocene palaeo sea-level markers in the Gulf of Naples. Alp. Mediterr. Quat.

[14] Biondo M., Buosi C., Trogu D., Mansfield H., Vacchi M., Ibba A., Porta M., Ruju A., De Muro S. (2020). Natural vs. Anthropic Influence on the Multidecadal Shoreline Changes of Mediterranean Urban Beaches: Lessons from the Gulf of Cagliari (Sardinia). Water.

[15] Revelles J., Ghilardi M., Rossi V., Currás A., López-Bultó O., Brkojewitsch G., Vacchi M. (2019). Coastal landscape evolution of Corsica island (W. Mediterranean): Palaeoenvironments, vegetation history and human impacts since the early Neolithic period. Quat. Sci. Rev..

[16] Khan N.S., Horton B.P., Engelhart S., Rovere A., Vacchi M., Ashe E.L., Törnqvist T.E., Dutton A., Hijma M.P., Shennan I. (2019). Inception of a global atlas of sea levels since the Last Glacial Maximum. Quat. Sci. Rev..

[17] Rovere A., Vacchi M., Parravicini V., Bianchi C., Zouros N., Firpo M. (2011). Bringing geoheritage underwater: Definitions, methods, and application in two Mediterranean marine areas. Environ. Earth Sci..

[18] Vacchi M., Ermolli E.R., Morhange C., Ruello M.R., Di Donato V., Di Vito M.A., Giampaola D., Carsana V., Liuzza V., Cinque A. (2020). Millennial variability of rates of sea-level rise in the ancient harbour of Naples (Italy, western Mediterranean Sea). Quat. Res..

[19] Di Luccio D., Benassai G., Di Paola G., Rosskopf C.M., Mucerino L., Montella R., Contestabile P. (2018). Monitoring and modelling coastal vulnerability and mitigation proposal for an archaeological site (Kaulonia, Southern Italy). Sustainability.

[20] Anfuso G., Postacchini M., Di Luccio D., Benassai G. (2021). Coastal sensitivity/vulnerability characterization and adaptation strategies: A review. J. Mar. Sci. Eng..

[21] Trobec A., Šmuc A., Poglajen S., Vrabec M. (2017). Submerged and buried Pleistocene river channels in the Gulf of Trieste (Northern Adriatic Sea): Geomorphic, stratigraphic and tectonic inferences. Geomorphology.

[22] Fontana A., Ronchi L., Rossato S., Mozzi P. (2018). Lidar-derived dems for geoarchaeological investigations in alluvial and coastal plains. Alp. Mediterr. Quat.

[23] Collin A., Ramambason C., Pastol Y., Casella E., Rovere A., Thiault L., Espiau B., Siu G., Lerouvreur F., Nakamura N. (2018). Very high resolution mapping of coral reef state using airborne bathymetric LiDAR surface-intensity and drone imagery. Int. J. Remote Sens..

[24] Ronchi L., Fontana A., Cohen K.M., Stouthamer E. (2021). Late Quaternary landscape evolution of the buried incised valley of Concordia Sagittaria (Tagliamento River, NE Italy): A reconstruction of incision and transgression. Geomorphology.

[25] Ronchi L., Fontana A., Correggiari A., Remia A. (2019). Anatomy of a transgressive tidal inlet reconstructed through high-resolution seismic profiling. Geomorphology.

[26] Castellanos-Galindo G.A., Casella E., Mejía-Rentería J.C., Rovere A. (2019). Habitat mapping of remote coasts: Evaluating the usefulness of lightweight unmanned aerial vehicles for conservation and monitoring. Biol. Conserv..

[27] Novak A., Šmuc A., Poglajen S., Vrabec M. (2020). Linking the high-resolution acoustic and sedimentary facies of a transgressed Late Quaternary alluvial plain (Gulf of Trieste, northern Adriatic). Mar. Geol..

[28] Casella E., Drechsel J., Winter C., Benninghoff M., Rovere A. (2020). Accuracy of sand beach topography surveying by drones and photogrammetry. Geo-Mar. Lett..

[29] Rovere A., Vacchi M., Firpo M., Carobene L. (2011). Underwater geomorphology of the rocky coastal tracts between Finale Ligure and Vado Ligure (western Liguria, NW Mediterranean Sea). Quat. Int..

[30] Plets R., Dix J., Bates R. (2013). Marine Geophysics Data Acquisition, Processing and Interpretation. http://www.english-heritage.org.uk/publications/marine-geophysics-data-acquisition-processing-interpretation/MGDAPAI-guidance-notes.pdf.

[31] Mattei G., Giordano F. (2015). Integrated geophysical research of Bourbonic shipwrecks sunk in the Gulf of Naples in 1799. J. Archaeol. Sci. Rep..

[32] Somma R., Iuliano S., Matano F., Molisso F., Passaro S., Sacchi M., Troise C., De Natale G. (2016). High-resolution morpho-bathymetry of Pozzuoli Bay, southern Italy. J. Maps.

[33] Nocerino E., Menna F., Remondino F. (2014). Accuracy of typical photogrammetric networks in cultural heritage 3D modeling projects. Int. Arch. Photogramm. Remote Sens. Spat. Inf. Sci..

[34] Nocerino E., Menna F. (2020). Photogrammetry: Linking the world across the water surface. J. Mar. Sci. Eng..

[35] Lo Brutto M., Dardanelli G. (2017). Vision metrology and Structure from Motion for archaeological heritage 3D reconstruction: A Case Study of various Roman mosaics. Acta Imeko.

[36] Neyer F., Nocerino E., Grün A. (2019). Image quality improvements in low-cost underwater photogrammetry. Int. Arch. Photogramm. Remote Sens. Spat. Inf. Sci..

[37] Drap P. (2012). Underwater photogrammetry for archaeology. Special Applications of Photogrammetry.

[38] Hamal S.N.G. (2023). Investigation of Underwater Photogrammetry Method: Challenges and Photo Capturing Scenarios of the Method. Adv. Underw. Sci..

[39] Hamal S.N.G., Ali U., Yiğit A.Y. (2021). Three-Dimensional Modeling of an Object Using Underwater Photogrammetry. Adv. Underw. Sci..

[40] Biyik M.Y., Atik M.E., Duran Z. (2023). Deep learning-based vehicle detection from orthophoto and spatial accuracy analysis. Int. J. Eng. Geosci..

[41] Dong S., Wang P., Abbas K. (2021). A survey on deep learning and its applications. Comput. Sci. Rev..

[42] Modasshir M., Rekleitis I. Augmenting coral reef monitoring with an enhanced detection system. Proceedings of the IEEE International Conference on Robotics and Automation.

[43] Fulton M., Hong J., Islam M.J., Sattar J. Robotic detection of marine litter using deep visual detection models. Proceedings of the 2019 International Conference on Robotics and Automation (ICRA).

[44] Beijbom O., Edmunds P.J., Kline D.I., Mitchell B.G., Kriegman D. Automated annotation of coral reef survey images. Proceedings of the 2012 IEEE Conference on Computer Vision and Pattern Recognition.

[45] Mattei G., Amato L., Caporizzo C., Cinque A., Pappone G., Sorrentino A., Stocchi P., Troisi S., Aucelli P.P. (2023). Reconstructing anthropic coastal landscape of Campi Flegrei volcanic area (Southern Italy) during the Roman period from multi-technique surveys. J. Maps.

[46] Ciaramella A., Perrotta F., Pappone G., Aucelli P., Peluso F., Mattei G. (2021). Environment object detection for marine argo drone by deep learning. Pattern Recognition, Proceedings of the ICPR International Workshops and Challenges, Virtual Event, 10–15 January 2021.

[47] Ren S., He K., Girshick R., Sun J. Faster r-cnn: Towards real-time object detection with region proposal networks. Proceedings of the Advances in Neural Information Processing Systems 28: Annual Conference on Neural Information Processing Systems 2015.

[48] Bingham B., Foley B., Singh H., Camilli R., Delaporta K., Eustice R., Mallios A., Mindell D., Roman C., Sakellariou D. (2010). Robotic tools for deep water archaeology: Surveying an ancient shipwreck with an autonomous underwater vehicle. J. Field Robot..

[49] Ødegård Ø., Sørensen A.J., Hansen R.E., Ludvigsen M. (2016). A new method for underwater archaeological surveying using sensors and unmanned platforms. IFAC-PapersOnLine.

[50] Mellone G., De Vita C.G., Sánchez-Gallegos D.D., Di Luccio D., Mattei G., Peluso F., Aucelli P.P.C., Ciaramella A., Montella R. A containerized distributed processing platform for autonomous surface vehicles: Preliminary results for marine litter detection. Proceedings of the 2023 31st Euromicro International Conference on Parallel, Distributed and Network-Based Processing (PDP).

